# Natural Killer Cell Phenotype and Functionality Affected by Exposure to Extracellular Survivin and Lymphoma-Derived Exosomes

**DOI:** 10.3390/ijms22031255

**Published:** 2021-01-27

**Authors:** Heather R. Ferguson Bennit, Amber Gonda, Janviere Kabagwira, Laura Oppegard, David Chi, Jenniffer Licero Campbell, Marino De Leon, Nathan R. Wall

**Affiliations:** 1 Division of Biochemistry, Department of Basic Science, Loma Linda University School of Medicine, Loma Linda, CA 92350, USA; hferguson@llu.edu (H.R.F.B.); ambergonda@gmail.com (A.G.); jkabagwira@students.llu.edu (J.K.); lauraoppegard@gmail.com (L.O.); David.Chi@KetteringHealth.org (D.C.); 2 Center for Health Disparities & Molecular Medicine, Department of Basic Science, Loma Linda University School of Medicine, 11085 Campus Street, Mortensen Hall 160, Loma Linda, CA 92350, USA; jlicerocampbell@students.llu.edu (J.L.C.); mdeleon@llu.edu (M.D.L.); 3 Division of Physiology, Department of Basic Science, Loma Linda University School of Medicine, Loma Linda, CA 92350, USA

**Keywords:** NK cells, survivin, exosome, granzyme, perforin

## Abstract

The inherent abilities of natural killer (NK) cells to recognize and kill target cells place them among the first cells with the ability to recognize and destroy infected or transformed cells. Cancer cells, however, have mechanisms by which they can inhibit the surveillance and cytotoxic abilities of NK cells with one believed mechanism for this: their ability to release exosomes. Exosomes are vesicles that are found in abundance in the tumor microenvironment that can modulate intercellular communication and thus enhance tumor malignancy. Recently, our lab has found cancer cell exosomes to contain the inhibitor of apoptosis (IAP) protein survivin to be associated with decreased immune response in lymphocytes and cellular death. The purpose of this study was to explore the effect of survivin and lymphoma-derived survivin-containing exosomes on the immune functions of NK cells. NK cells were obtained from the peripheral blood of healthy donors and treated with pure survivin protein or exosomes from two lymphoma cell lines, DLCL2 and FSCCL. RNA was isolated from NK cell samples for measurement by PCR, and intracellular flow cytometry was used to determine protein expression. Degranulation capacity, cytotoxicity, and natural killer group 2D receptor (NKG2D) levels were also assessed. Lymphoma exosomes were examined for size and protein content. This study established that these lymphoma exosomes contained survivin and FasL but were negative for MHC class I-related chains (MIC)/B (MICA/B) and TGF-β. Treatment with exosomes did not significantly alter NK cell functionality, but extracellular survivin was seen to decrease natural killer group 2D receptor (NKG2D) levels and the intracellular protein levels of perforin, granzyme B, TNF-α, and IFN-γ.

## 1. Introduction

The active role of the tumor microenvironment (TME) in tumor pathogenesis has recently been gaining intense scrutiny as it can be responsible for delivering signals for clonal expansion [1], drug resistance [2], metastatic migration [3], and immune modulation [4]. Cancer development is not an autonomous process but depends on surrounding nonmalignant components such as fibroblasts, microvasculature, stroma, mesenchymal cells, and immune cells [5,6]. Interaction between tumors and their surrounding environment occurs via direct contact, soluble factors, and material exchange [7]. One important method of cell-to-cell interaction that has received much interest in the preceding decade for communication and signaling is through information transfer between cells via small bilayer lipid vesicles known as exosomes, or more generally, as extracellular vesicles (EVs). These endosomally derived 30–150 nm vesicles contain proteins, lipids, and nucleic acids such as miRNA, mRNA [8,9], and even DNA [10,11] that can be sent into the extracellular environment and delivered to recipient cells either locally or systemically. Most tissue and immune cell types have been shown to produce exosomes, but tumor cells seem to produce increased numbers [12,13,14,15]. These tumor-derived exosomes (TEX) have a myriad of effects within the TME and beyond, with resulting enhanced malignant potential, niche preparation for metastasis, and angiogenesis [16,17]. Importantly, TEX can have many influences on immune cells, resulting in both activation and suppression of tumor immunity [18,19,20,21,22].

Natural killer (NK) cells are important in the surveillance and eradication of virally infected and malignantly transformed cells and are susceptible to suppression in the TME [23,24]. TEX carrying ligands such as FasL, TGF-β, IL-10, and natural killer group 2D receptor ligands (NKG2DL) are implicated in the downregulation of T cell and NK cell activity [25,26,27,28,29,30]. As NK cells form the first line of defense and are ready to kill without prior activation, their function is critical to controlling spontaneous tumorigenesis of epithelial and lymphoid malignancies [31,32]. NK cell activation is based on a complex interplay of their inhibitory and activating receptors. Healthy cells display MHC class I proteins on their surface, which bind to inhibitory KIRs (killer-cell immunoglobulin-like receptors) on NK cells to prevent activation. Stressed cells often lose MHC class I and upregulate ligands such as MICA/B (MHC class I-related chains (MIC) A/B) and ULBPs (UL16 binding proteins), which bind to activating receptor NKG2D (natural killer group 2D receptor) [33,34,35]. Ligand binding stimulates downstream signaling to activate cytokine production of IFN-γ and TNF-α and degranulation to release cytolytic proteins such as perforin and granzyme B. These proteins initiate apoptosis in the target cell by opening pores in the target cell membranes and cleaving cellular proteins. The cytolytic functionality of NK cells can be inhibited by TEX through decreased release of perforin [36] and overexposure to soluble MICA/B with subsequent downregulation of the NKG2D receptor [18,30,37,38,39].

Exosomes, as a vehicle for immune suppression, can act through multiple modalities, such as surface ligands such as FasL, PD-L1, and TGF-β, and content such as miRNAs and proteins [40]. In our lab, we discovered tumor-derived exosomes contain survivin [41,42], an anti-apoptotic protein that is generally found in the cytoplasm, mitochondria, and nucleus of cancer cells [43]. When this cancer-specific member of the inhibitor of apoptosis (IAP) family of proteins is found in the extracellular compartment, we have shown evidence of increased resistance to treatment, invasive potential, and proliferative capacity in surrounding cells [44]. Additionally, extracellular survivin can act upon T cells to skew their behavior toward Th2 and decrease CD8+ T cell activity [45]. It is unclear whether other immune cell populations, such as NK cells, are also able to be affected by survivin or exosomes from lymphoma cells. In this study, we investigated the influence of exosomes from lymphoma cell lines and survivin on NK cell function. Our results indicate that conditioning of NK cells by extracellular survivin-containing exosomes had little effect on NKG2D receptor levels, degranulation capacity, or cytolytic potential. However, while mRNA levels of NK cell proteins were not consistently affected, protein levels of IFN-γ, TNF-α, perforin, and granzyme B were decreased as a result of treatment with recombinant survivin protein.

## 2. Materials and Methods

### 2.1. Cell Culture

Human lymphoma cell lines WSU-DLCL2 and WSU-FSCCL were a kind gift from Ayad Al-Katib at Wayne State University (Detroit, MI, USA) [46]. Cell lines were grown in RPMI 1640 media supplemented with 10% USDA-sourced heat-inactivated fetal bovine serum (FBS) (Mediatech, Manassas, VA, USA), 4 mM L-glutamine, 0.1 mg/mL streptomycin, and 100 units/mL penicillin and incubated at 37 °C and 5% CO_2_. Trypan blue staining was used to measure cell density (confluent at 1 × 10^6^/mL) and viability (above 90%).

### 2.2. NK Cell Isolation and Culture

Natural killer (NK) cells were prepared from freshly collected plasma apheresis peripheral blood obtained from healthy donors at the Life Stream Blood Bank (San Bernardino, CA, USA), in accordance with Loma Linda University IRB protocols. Peripheral blood was prepared as described previously [47]. NK cells were obtained from the total lymphocyte fraction by negative immunomagnetic cell separation using either EasySep or RosetteSep negative selection kit (STEMCELL, Vancouver, BC, USA) or MACS magnetic NK cell isolation kit (Miltenyi Biotec, Auburn, CA, USA). The purity of the NK cell population was determined by flow cytometric analysis and ranged from 85–95%, with all kits showing comparable performance in obtaining CD3-/CD56+ NK cells (greater than 90% purity and viability). NK cells were cultured at a high density of 5 × 10^6^ cells/well in 6-well non-pyrogenic polystyrene culture plates overnight in RPMI 1640 media (Mediatech Inc. Manassas, VA, USA), supplemented with 10% FBS (Hyclone, Logan, UT, USA), 1 mM L-glutamine, 100 U/mL penicillin, 100 µg/mL streptomycin, and 100 U/mL human recombinant IL-2 before use in experiments.

### 2.3. Exosome Isolation and Characterization

Exosomes were isolated and characterized in accordance with the minimal information for studies of extracellular vesicles 2018 (MISEV2018) [48]. In short, MISEV2018 directs that both the source and the EV preparation be described quantitatively. Lymphoma cells were cultured for 24–48 h in media depleted of exosomes from fetal bovine serum (FBS, Hyclone, Logan, UT, USA) by overnight ultracentrifugation at 100,000× *g*. Exosomes were isolated using previously described methods [42] from conditioned medium after serial centrifugation with commercially available ExoQuick-TC (System Biosciences, Mountain View, CA, USA). Exosome pellets were resuspended in 40–70 μL PBS and either used immediately for experiments or stored at −80 °C. The quantity of exosomal protein was determined by BCA protein assay (Pierce/Thermo Scientific, Rockford, IL, USA). The size and concentration of exosomes isolated using ExoQuick were determined using nanoparticle tracking analysis (NTA) with a NanoSight NS300 instrument (NanoSight, Malvern Instruments Ltd., Malvern, UK). Samples were diluted 1:100 in PBS. Five sequential measurements of 60 s each were recorded at a flow rate of 20 µL/minute. The instrument was set to a detection threshold of 8 and 25 frames per second. Analysis was performed on software NTA 3.2 Dev Build 3.2.16 as we have previously reported [49]. In addition, and in compliance with MISEV2018, isolates were analyzed by Western blot and flow cytometry to determine if isolates carried three or more EV-associated proteins.

### 2.4. Flow Cytometry Antibodies

Antibodies and other reagents used were: Intracellular Fixation and Permeabilization Buffer kit, fixable viability dye e-Fluor 780, CD56 PerCP-eFluor 710, MICA/B Alexa Fluor 488, CD69 PE-Cy7 (eBioscience, Grand Island, NY, USA); CD16 PE, CD56 PE (BD Biosciences, San Jose, CA, USA); CD3 FITC, IFN-γ PE-Vio770, TNF-α PE-Vio770, CD107a FITC (Miltenyi Biotec, Auburn, CA, USA); CD3 PE-Cy7, CD3 FITC, CD56 APC (MY31) (Tonbo Bioscience, San Diego, CA, USA); perforin Pacific Blue, granzyme B Pacific Blue, NKG2D APC (BioLegend, San Diego, CA, USA); and survivin Alexa Fluor 647 (Cell Signaling Technology, Danvers, MA, USA). Data were collected on a Miltenyi MACSQuant Analyzer flow cytometer (Miltenyi Biotec, Auburn, CA, USA) and analyzed using FlowJo v10.0.8p software (Tree Star, Ashland, OR, USA).

### 2.5. Flow Cytometry Analysis of Exosomes

Purified exosomes (200 μg) were incubated with 10 μL of 4-μm-diameter aldehyde/sulfate latex beads (Invitrogen, Eugene, OR) in PBS for 1 h at room temperature with gentle agitation. After washing with PBS, samples were blocked with 200 mM glycine for 30 min and washed again. Beads were incubated with the following exosome-associated antibodies for 30 min: anti–CD63 (Millipore, Billerica, MA, USA), anti-CD9 (BD Biosciences, San Jose, CA, USA), anti-HLA-A,B,C (BioLegend, San Diego, CA, USA), anti-TSG101 (Santa Cruz Biotechnology, Santa Cruz, CA, USA), anti-HSP70 (Novus Biologicals, Littleton, CO, USA), anti-lysosomal membrane glycoproteins CD107a (LAMP-1) (Miltenyi Biotec, Auburn, CA, USA), anti-MICA/B (eBioscience, Grand Island, NY, USA), anti-CD81 (ProSci, Fort Collins, CO, USA), anti-TGF-β (Peprotech, Rocky Hill, NJ, USA), and anti-FasL (Boster, Pleasanton, CA, USA). Staining with secondary antibodies for 30 min followed, using goat anti-rabbit Alexa Fluor 488 (Invitrogen, Eugene, OR, USA) and goat anti-mouse FITC (Becton Dickinson, Franklin Lakes, NJ, USA). A “bead-only” control as well as isotype-matched antibody controls were also prepared. Samples were washed twice, fixed with 1% paraformaldehyde and analyzed using a MACSQuant Analyzer and FlowJo software. Single beads were gated for fluorescence analysis.

### 2.6. Western Blot

Western blotting was performed as previously described [42]. Membranes were immunostained using the following antibodies: rabbit polyclonal anti-survivin (1:500–1000, Novus Biologicals, Littleton, CO, USA); rabbit monoclonal anti-XIAP, anti-cIAP1, anti-cIAP2, rabbit polyclonal anti-β-actin (1:500–1000, Cell Signaling Technology, Danvers, MA, USA); mouse polyclonal anti-LAMP-1 (1:500, BioLegend, San Diego, CA, USA); mouse monoclonal anti-granzyme B (OriGene, Rockville, MD, USA); and rabbit polyclonal anti-perforin (Boster, Pleasanton, CA, USA). Goat anti-mouse and goat anti-rabbit Dylight 800 secondary antibodies (Thermo Scientific, Waltham, MA, USA) were used at a 1:10,000 dilution. Immunoreactive bands were detected using the Odyssey imaging system (LI-COR Biosciences, Lincoln, NE, USA). β-actin or lysosomal membrane glycoproteins CD107a (LAMP-1) were used as loading controls for either cell lysates or exosomal protein, respectively.

### 2.7. Degranulation Assay

NK cell degranulation was evaluated by staining for CD107a (LAMP-1) during NK cell stimulation. Briefly, peripheral blood mononuclear cells (PBMCs) were treated for 24 h either with or without IL-2 (100 U/mL) and exposed to either 0, 0.1, or 1.0 µg ml of recombinant survivin (Abcam, Cambridge, MA). Cells were then incubated in 96-well round bottom plates for 6 h with phorbol-12-myristate-13-acetate (PMA) (10 ng/well) and ionomycin (50 ng/well) as a positive control for activation, K562 cells (5 × 10^5^/well) for targeted stimulation, or media as a negative control. Monensin (3 µM, Sigma Aldrich, St. Louis, MO, USA) was added after the first hour to inhibit Golgi transport and prevent both release of cytokines (for intracellular detection) and to prevent acidification of endocytosed vesicles containing CD107a antibody. The anti-CD107a-FITC antibody (Miltenyi Biotec, Auburn, CA, USA) or isotype control were added before NK cells were stimulated to allow binding to the degranulation marker as granules were exocytosed. Surface and intracellular staining was performed prior to flow cytometry and analysis performed on the CD3-/CD56+ gated population.

### 2.8. NKG2D Receptor Expression

Following the protocols previously published (Hong 2014), PBMCs obtained from healthy volunteers were co-incubated with isolated exosomes or survivin to determine the percentage of NK cells (CD3-/CD56+) expressing the natural cytotoxicity receptor NKG2D on the cell surface. Briefly, PBMCs were activated with IL-2 alone or with additional stimulation using PMA/ionomycin or K562 cells (human immortalized myelogenous leukemia cells). PBMCs were co-incubated with (10 µg protein/mL) exosomes or survivin (0–1 µg/mL) for 24 h at 37 °C and then stained with APC-conjugated NKG2D antibody (BioLegend, San Diego, CA, USA) and run on a MACSQuant flow cytometer (Miltenyi Biotec, Auburn, CA, USA). The frequency of NKG2D positive cells was measured from the gated CD3 negative/CD56 (positive) NK cells. The data are expressed as percent NKG2D positive cells/total CD3 (negative) CD56 (positive) cells. The mean fluorescence intensity (MFI) of NKG2D on the NK cells was also recorded. PBMCs that were incubated in medium without exosomes were used as controls.

### 2.9. Cytotoxicity Assay

To determine cell-specific killing capacity of NK cells, the release of cytoplasmic lactate dehydrogenase (LDH) from lysed target K562 (erythroleukemia) cells was measured using the CytoTox 96 non-radioactive cytotoxicity assay (Promega, Fitchburg, WI, USA) according to the manufacturer’s instructions. Briefly, NK cells isolated from healthy donors were incubated overnight at 5 × 10^6^/mL in complete RPMI1640 media with 100 IU/mL IL-2. NK cells were then treated for 24 h with either 10 µg/mL survivin protein (Abcam, Cambridge, MA, USA), exosomes from lymphoma cells (10–50 µg/mL), or media for control. A range of effector to target ratios from 20:1, 10:1, 5:1, 2.5:1, 1.25:1, 0.625:1 was used, and the target K562 cells were added at 10,000 cells/well. After 4 h of incubation at 37 °C, the LDH-containing media was transferred to a separate plate and absorbance read at 490 nm with a Bio-Tek µQuant microplate reader. The percentage of specific lysis was calculated according to a standard equation, specific lysis (%) = (experimental release − effector spontaneous release − target spontaneous release)/(target maximum release − target spontaneous release) × 100.

### 2.10. Polymerase Chain Reaction (PCR)

Perforin, granzyme B, TNF-α, and IFN-γ mRNA expression was evaluated by PCR from magnetically sorted NK cells treated either with survivin protein or lymphoma-derived exosomes or an untreated control. Cells were resuspended in 500 µL TRI reagent solution (Ambion, Grand Island, NY, USA), and RNA was extracted according to the manufacturer’s protocol. RNA concentration was measured using Nanodrop 2000c (Thermo Fisher Scientific, Waltham, MA, USA). Single-strand cDNA was synthesized using High Capacity cDNA Reverse Transcription Kit (Applied Biosystems, Foster City, CA, USA) according to manufacturer’s instructions and its concentration measured using the Nanodrop 2000c (Thermo Fisher Scientific, Waltham, MA, USA). PCR amplification was performed with Thermo Scientific Maxima Hot Start PCR Master Mix (2X) (Thermo Scientific, Grand Island, NY, USA) using 200 ng of cDNA. PCR thermal cycles were performed with Eppendorf Mastercycler gradient thermal cycler using the following conditions: 2 min at 94 °C, followed by 34–38 cycles of 30 s at 95 °C, 45 s at annealing melting temperature ™, and 1 min at 72 °C, followed by a final extension time of 10 min at 72 °C. The primer pairs were designed with Primer-BLAST (National Center for Biotechnology Information (NCBI)) and synthesized by IDT (Integrated DNA Technologies, San Diego, CA, USA). Sequences for each primer pair are listed in Table 1. Glyceraldehyde-3-phosphate dehydrogenase (GAPDH) housekeeping gene was used as control. The PCR products were analyzed on a 1% agarose gel by electrophoresis in the presence of ethidium bromide and visualized with UV light.

### 2.11. Real-Time qRT-PCR Analysis

NK cell RNA was prepared as described previously. Target genes perforin, granzyme B, IFN-γ, and TNF-α were quantified by real-time PCR using the CFX96 system (Bio-Rad Laboratories, Hercules, CA, USA). GAPDH was used as the reference gene for normalization. Reactions were performed in triplicate with a 25-μL mixture containing cDNA samples, primers, and iQ Sybr Green supermix (Bio-Rad Laboratories, Hercules, CA, USA). The relative amount of mRNA in experimental cells was calculated using the 2−ΔΔCT method and CFX manager software (Bio-Rad Laboratories, Hercules, CA, USA).

### 2.12. Statistical Analysis

Data are expressed as the mean ± SE (standard error). GraphPad Prism software v.5.01 for Windows (San Diego, CA, USA) was used to conduct statistical analysis. A Student’s *t*-test and one-way ANOVA was used, with post hoc Dunnett’s multiple comparison test for the comparisons between groups. Statistical significance was indicated by a probability of *p* < 0.05. Each experiment was repeated at least three times to assess the level of reproducibility.

## 3. Results

**Figure 1:**
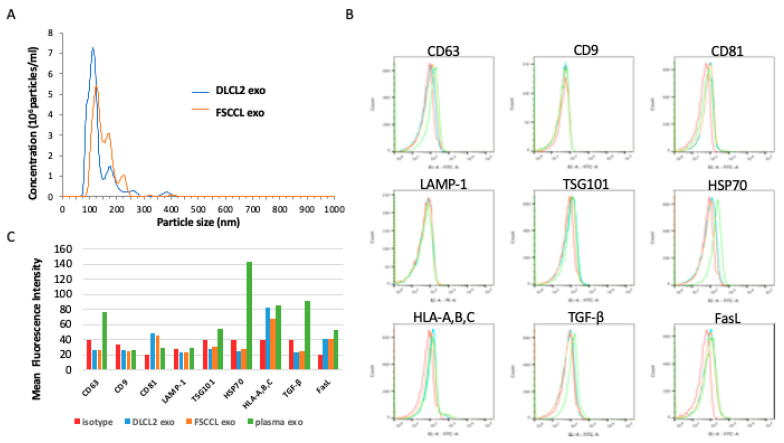
Extracellular vesicles (EVs) derived from WSU-DLCL2 and WSU-FSCCL lymphoma cell lines are exosomes. (**A**) Sizing analysis of lymphoma EVs was performed with the NanoSight NS300. Mean size for exosomes was 137 nm (+/− 56 nm) for DLCL2 and 156 nm (+/− 63 nm) for FSCCL. (**B**) For analysis of expression of surface molecules, 200 μg of exosomes was incubated with 10 μL of 4-μm-diameter aldehyde/sulfate latex beads and stained with antibodies for EVs: CD63, CD9, CD81, lysosomal membrane glycoproteins CD107a (LAMP-1), TSG101, HSP70, HLA-A,B,C, TGF-β, and FasL. Only the population containing single beads was gated and analyzed. Red = isotype, green = plasma exosomes, blue = DLCL2 exosomes, and orange = FSCCL exosomes. (**C**) Mean fluorescence intensity (MFI) of exosome staining was compared with exosomes stained with an isotype control and exosomes from plasma. A “bead-only” control, as well as isotype-matched antibody controls were also prepared. Samples were washed twice, fixed with 1% paraformaldehyde, and analyzed using a MACSQuant Analyzer and FlowJo software.

Isolated exosomes were evaluated for size using nanoparticle tracking analysis (NTA) with a NanoSight NS300 (Malvern Instruments Ltd., Malvern, UK). Mean DLCL2 vesicle diameter was 137 nm (+/− 56 nm), with a mode of 114 nm. Mean FSCCL vesicle size was 156 nm (+/− 63 nm), with a mode of 128 nm (Figure 1A). We also evaluated the lymphoma exosomes for several protein markers using flow cytometry and Western blotting. Our data showed these lymphoma vesicles did not strongly express CD63, LAMP-1, CD9, or TSG101. However, there was detection of MHC class I and CD81. As a control, protein expression from exosomes derived from normal human plasma were used. These plasma vesicles were positive for CD63, TSG101, HSP70, and MHC class I (Figure 1B,C).

**Figure 2:**
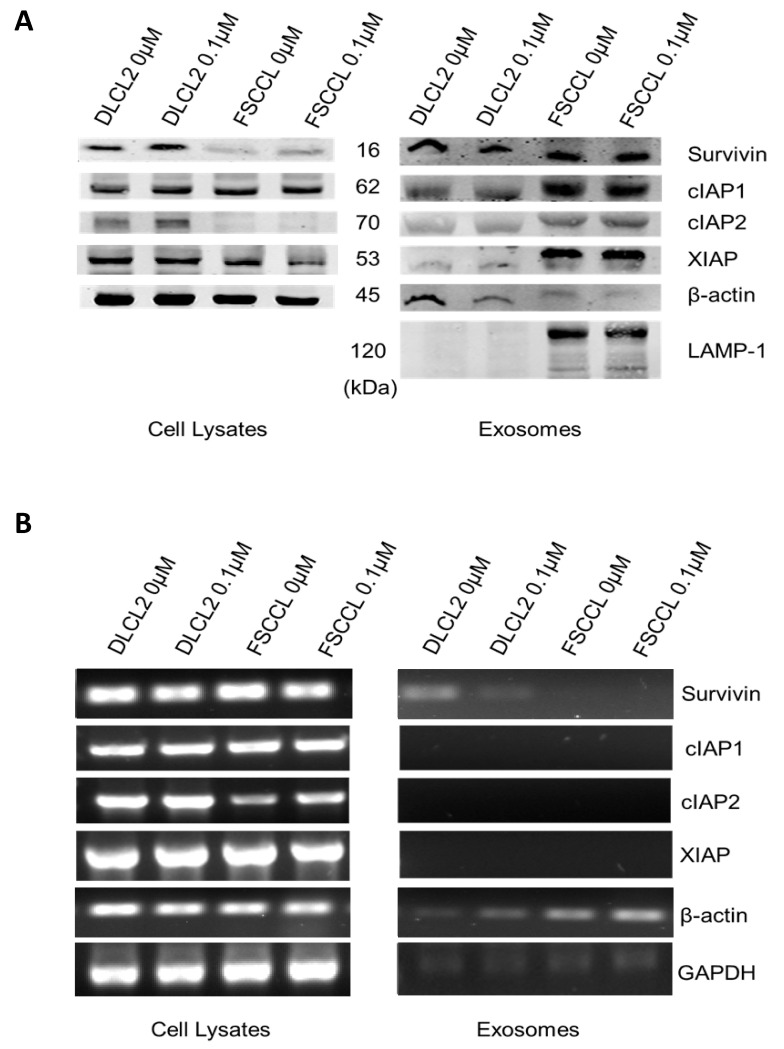
Lymphoma cells and exosomes contain survivin and other inhibitor of apoptosis (IAP) proteins. Cells were treated with sublethal amounts of etoposide (0.1 μM) for 24 h in order to determine whether stress would change the IAP localization. (**A**) Western blots (30 mg protein) show the IAPs survivin, cIAP1, cIAP2, and XIAP are contained in cell lysates (left) and exosomes (right). GAPDH and β-actin were used as controls. (**B**) RT-PCR analysis of mRNA (200 ng) content in cells and exosomes.

It was previously established that exosomes from an aggressive diffuse large cell lymphoma (WSU-DLCL2) cell line contained survivin [42]. We wanted to confirm the presence of exosomal survivin in WSU-FSCCL, an indolent follicular small cleaved cell lymphoma cell line. Western blot confirmed that these B cell lymphoma cell lines expressed cellular and exosomal survivin, in addition to other IAPs (Figure 2A). It was also determined that sublethal amounts of stress due to treatment with etoposide (0.1 µM) did not alter IAP expression levels. PCR results were inconsistent in what IAPs were detectable, and often negative. Housekeeping genes β-actin and GAPDH were used as loading controls (Figure 2B).

Survivin in the extracellular space has been previously shown to be taken up by T cells and elicit effects such as decreased proliferation, decreased cytolytic capabilities of CD8+ T cells, and a skewing of the cytokine profile to that of a Th2 population [45]. As NK cells are frequently found to be inhibited in the TME, we investigated whether extracellular survivin or lymphoma exosomes containing survivin would also modify NK cell function.

**Figure 3:**
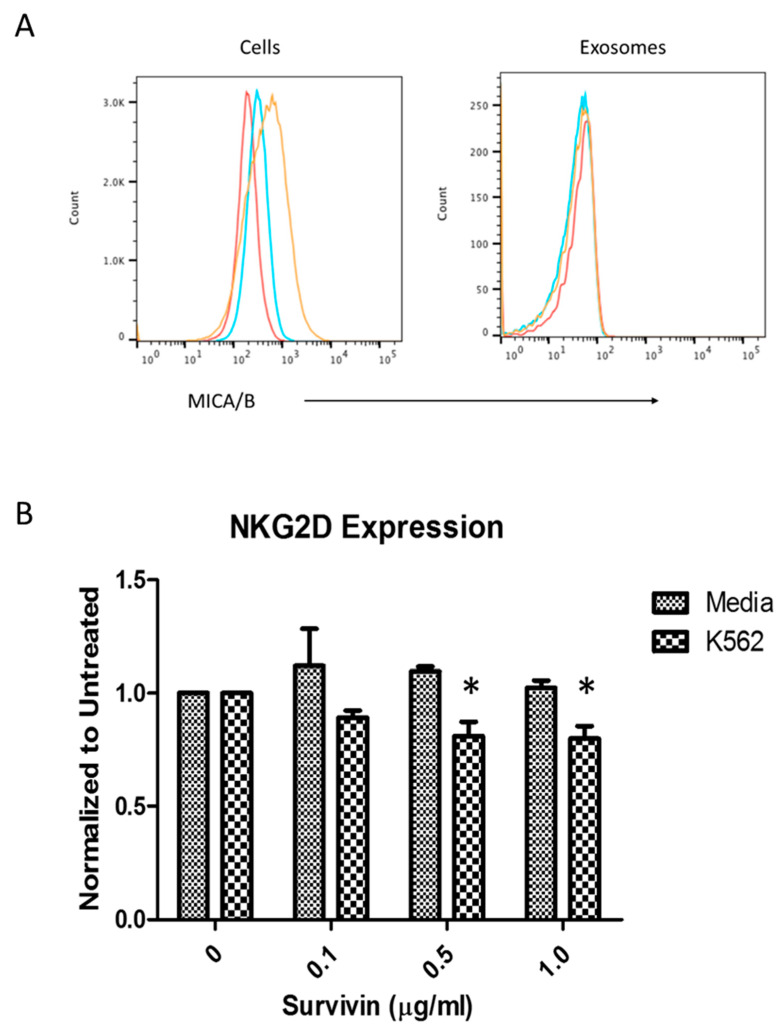
Natural killer group 2D receptor ligands (NKG2DL) were present on lymphoma cells but not exosomes, and treatment with survivin protein decreased natural killer group 2D receptor (NKG2D) expression. (**A**) Flow cytometry indicates that lymphoma cells expressed MHC class I-related chains (MICA)/B (an NKG2DL), but exosomes from these two lymphoma cell lines did not. Red = isotype, blue = DLCL2, orange = FSCCL. (**B**) NKG2D expression on natural killer (NK) cells (CD56+) after treatment with survivin (0–1.0 μg/mL). NK cells were activated by IL-2 (100 U/mL) and K562 (5 × 10^5^/well) target cells and treated with survivin for 24 h. The receptor expression was assessed by mean fluorescence intensity (MFI) measured by fluorescent staining with NKG2D–APC antibody. MFI was normalized to an untreated group and data are presented as mean (±SEM) of three independent experiments (* is *p* < 0.05), survivin treatment versus untreated control. Data were collected on a Miltenyi MACSQuant Analyzer flow cytometer and analyzed using FlowJo v10.0.8p software.

A well-recognized mechanism by which NK cells are inhibited in the TME is through interference with the activating receptor NKG2D. Soluble and exosomal release of the NKG2D ligand MICA/B has been shown to reduce NKG2D expression on NK cells [50]. We therefore examined whether lymphoma exosomes carried MICA/B (Figure 3) and if exposure to these exosomes or extracellular survivin protein would alter NKG2D levels. Analysis of exosome-coated aldehyde beads by flow cytometry did not detect expression of MICA/B, although the parent lymphoma cells did express this NKG2D ligand (Figure 3A). Investigation of NKG2D levels on NK cells did not find significant changes after exposure to exosomes or survivin, except in the NK cell group activated by a classical target, erythroleukemia cell line K562 [51]. In this group, there was a noticeable decrease in NKG2D levels after treatment with survivin protein, compared to media control (Figure 3B), although the mechanism by which this occurred is yet to be determined.

**Figure 4:**
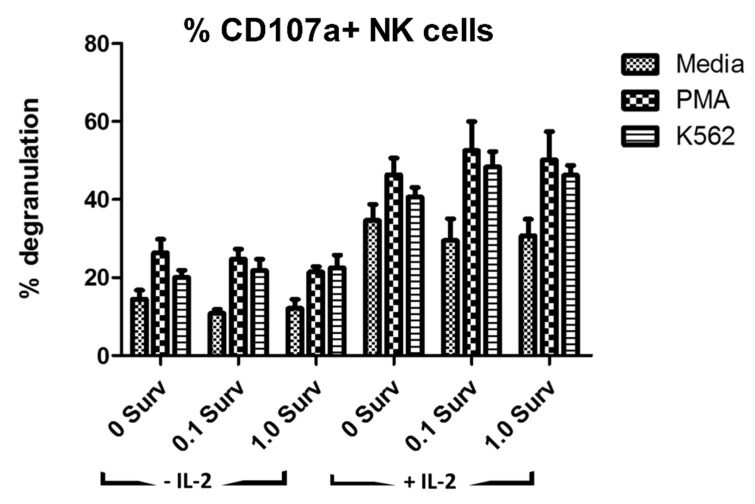
Degranulation of NK cells affected by IL-2 and activation by PMA and K562, but not by survivin. Peripheral blood mononuclear cells (PBMCs) from healthy donors (*n* = 7) were treated with recombinant survivin protein (0–1.0 μg/mL) with or without IL-2 (100 U/mL) for 36 h. Degranulation activity of the gated CD56+ NK cell population was measured after 6 h of stimulation by PMA (10 ng/well) and ionomycin (50 ng/well) as positive control, K562 target cells (5 × 10^5^/well), or media control using flow cytometry. Data presented as % of total gated NK cells that were CD107a-FITC +. For flow cytometry, data were collected on a Miltenyi MACSQuant Analyzer flow cytometer and analyzed using FlowJo v10.0.8p software.

To evaluate whether mechanisms other than NKG2D downregulation might be contributing to inhibition of NK cell function, we tested degranulation levels of NK cells after conditioning with survivin or lymphoma exosomes. Activated NK cells mobilize cytosolic granules containing perforin and granzyme B to the plasma membrane for release, or degranulation. This process exposed the lysosomal membrane glycoproteins CD107a, also known as LAMP-1, which can be used as a measurement of NK cell activation or inhibition [52]. As expected, there were higher levels of degranulation in the NK cell group activated with IL-2 and increased degranulation in NK cells that were activated with PMA/ionomycin and K562 cells. However, no significant changes were seen upon treatment with survivin (Figure 4) or exosomes (data not shown).

**Figure 5:**
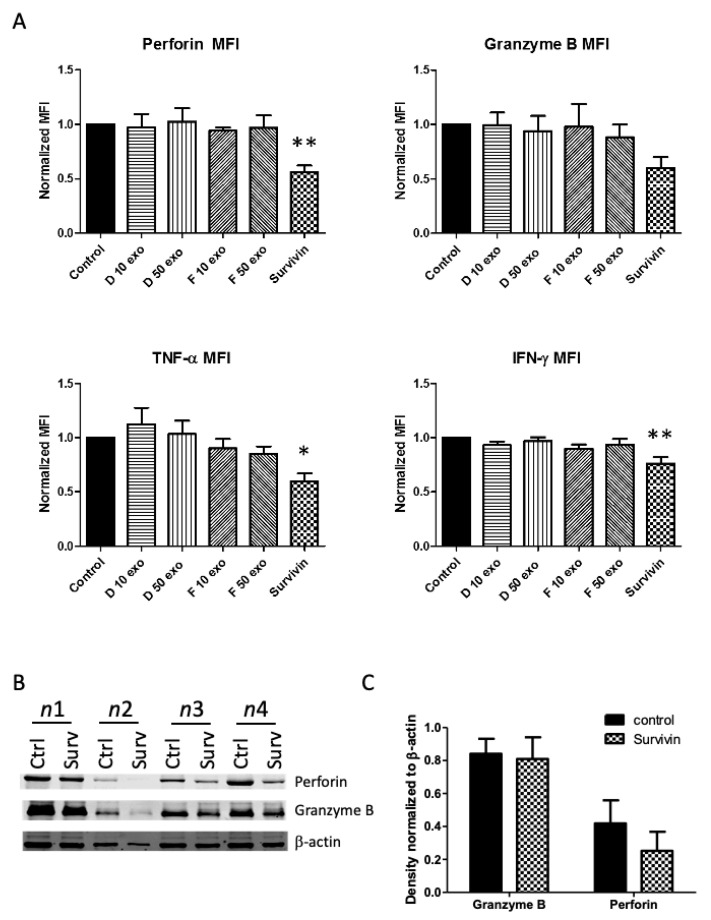
Survivin decreased NK cell intracellular protein levels of perforin, granzyme B, TNF-α, and IFN-γ. (**A**) CD3-, CD56+ NK cells were treated with extracellular survivin protein (0–1.0 μg/mL) or lymphoma exosomes (10–50 µg/mL) for 24 h. Expression of perforin, granzyme B, TNF-α, and IFN-γ was measured by intracellular flow cytometry (*n* = 5). Two concentrations of exosomes were investigated (10 μg and 50 μg). MFI was normalized to an untreated group and data are presented as mean (±SEM) of five independent experiments (* is *p* < 0.05, ** is *p* < 0.01), survivin treatment versus untreated control. (**B**) Western blot analysis, in order to determine protein levels of perforin and granzyme B, was conducted (x4) from NK cells after treatment with survivin. (**C**) Densitometry by ImageJ with b-actin normalization (*n* = 4).

NK cell function can also be affected by the levels of its cytotoxic granule proteins and cytokines, as seen, for example, in the TME of multiple myeloma, where inhibition of NK cell activity occurred via lower levels of perforin and granzyme B, despite unchanged degranulation [53]. Therefore, we next determined whether levels of perforin, granzyme B, or cytokines TNF-α or IFN-γ would be affected by exposure of NK cells to lymphoma exosomes or survivin protein. We treated NK cells with either exosomes (10 or 50 μg/mL) or survivin protein and investigated protein levels by intracellular flow cytometry (Figure 5A). Protein levels were not affected by activation with PMA/ionomycin or K562 activation (data not shown). Exosomes were not able to exert a noticeable effect upon intracellular protein levels. However, treatment with extracellular survivin protein consistently decreased protein amounts. We also used Western blot analysis to compare protein levels of perforin and granzyme B in NK cells treated with survivin protein (Figure 5B).

**Figure 6:**
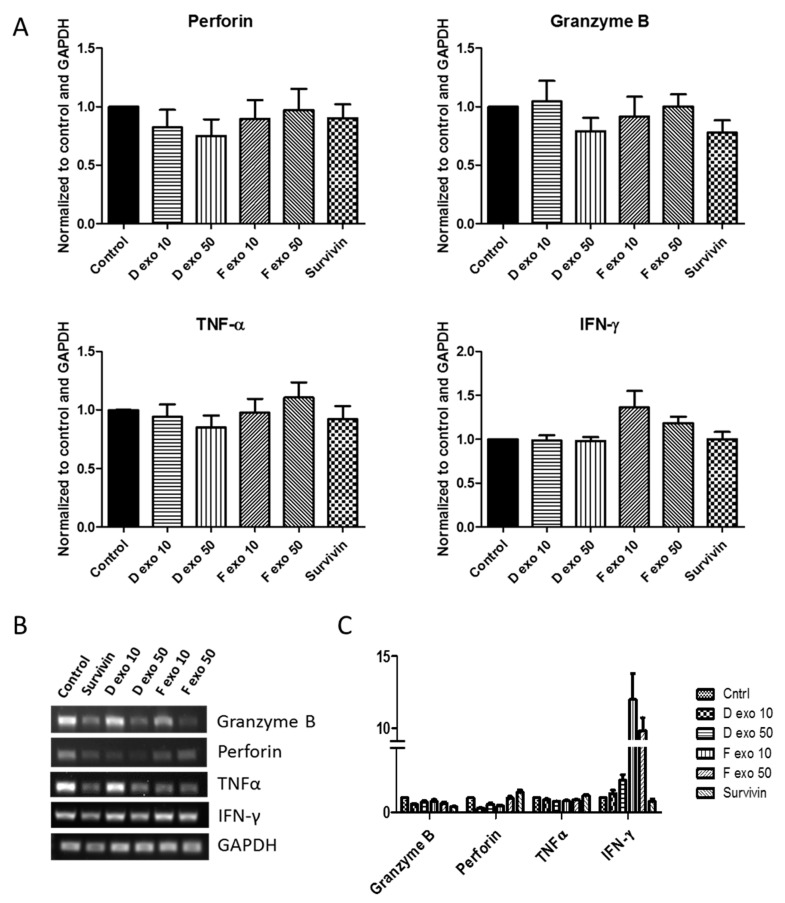
Lymphoma exosomes and survivin had no consistent effect on mRNA levels of NK cell cytokines and granules. (**A**) ImageJ density analysis of block RT-PCR bands did not show statistical significance in mRNA levels after 24 h treatment with lymphoma exosomes (10 μg and 50 μg) or survivin (*n* = 5). (**B**) Block RT-PCR amplicon products from NK cells from one donor run on 1% agarose gel. (**C**) Gene expression (ΔΔCq) was measured using real time qRT-PCR performed with a Bio-Rad CFX (*n* = 5) and showed the greatest degree of change in IFN-g.

In addition to measuring protein levels, we also investigated mRNA expression of perforin, granzyme B, TNF-α, and IFN-γ. We found that treatment of NK cells with lymphoma exosomes or extracellular survivin for 24 h did not result in consistent alterations to levels of RNA by either block RT-PCR (Figure 6A) or real time qRT-PCR (Figure 6C).

**Figure 7:**
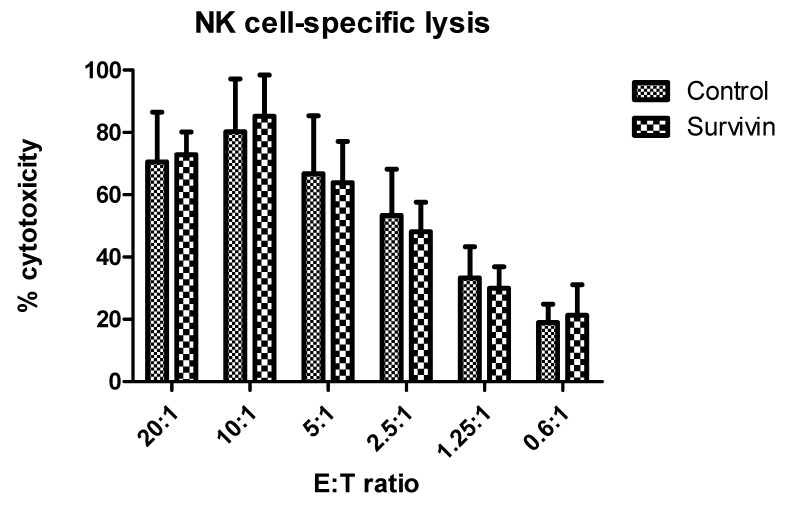
Survivin treatment had no effect on NK cell cytotoxicity. To determine cell-specific killing capacity of NK cells, the release of cytoplasmic lactate dehydrogenase (LDH) from lysed target K562 (erythroleukemia) cells (5 × 10^5^/well) was measured using the CytoTox 96 non-radioactive cytotoxicity assay. Serial dilutions of the effector to target (E:T) ratio from 20:1 to 0.625:1. NK effector cells were activated overnight with IL-2 (100 U/mL) and treated with 10 μg/mL survivin for 24 h before being co-incubated with K562 cells for 4 h. Upon conclusion of the incubation at 37°C, the LDH-containing media was transferred to a separate plate and absorbance read at 490 nm with a Bio-Tek µQuant microplate reader. The percentage of specific lysis was calculated according to a standard equation, specific lysis (%) = (experimental release − effector spontaneous release − target spontaneous release)/(target maximum release − target spontaneous release) × 100.

As a test of functionality, we investigated the cytotoxic potential of NK cells by measuring their ability to lyse K562 target cells. NK cells were treated with 10 μg/mL recombinant survivin for 24 h prior to performing the lactate dehydrogenase release assay. NK effector cells were mixed with K562 target cells at various ratios, and the percentage of specific lysis was calculated. As shown in Figure 7, exposure to survivin did not alter NK cell cytotoxic potential.

## 4. Discussion

The ability of tumors to avoid immune attack is critical for cancer development and metastasis, but the mechanisms responsible have yet to be fully elucidated. Cancer exosomes have emerged as mediators of immune suppression, facilitating tumor survival. In this work, we studied the influence of lymphoma-derived exosomes and extracellular survivin on NK cells. We hypothesized that tumor-derived exosomes containing survivin would affect NK functionality similar to previous findings concerning T cell suppression by extracellular survivin [45]. However, through investigating the EVs derived from lymphoma cells, we found that although survivin and other IAPs are present, there was little observable effect on NK cell functionality after exposure to these vesicles. Conversely, extracellular survivin treatment did decrease NK cell intracellular protein expression of perforin, granzyme B, TNF-α, and IFN-γ. This is perhaps due to a concentration effect, as the mixed contents of exosomes may dilute or overpower the influence of survivin delivered exosomally, as opposed to treatment by pure protein. While an effect on NK cell protein was seen with survivin by itself, the effect was lost by dilution into exosomes. Therefore, survivin in the TME sourced from necrosing tumor cells may show more effects than exosomal survivin due either to concentration or uptake issues, as NK cells have not been shown to efficiently uptake exosomes [47].

The lack of effects on NK cell functionality after exposure to lymphoma-derived vesicles could result from several factors. For example, lymphoma exosomes may not contain sufficient quantities of modulating proteins. Examination by Western blot and flow cytometry showed very low amounts of FasL, TGF-β, or MICA/B associated with these vesicles. There is literature support for NK cells being affected by tumor exosomes that harbor these proteins [54]. Ovarian exosomes from cell lines and patients have shown variable surface expression of NKG2DL (MICA/B, ULBP1-3), with concomitant ability to downregulate NKG2D on PBMCs, decrease degranulation, and inhibit cytotoxicity [55]. Although leukemia and lymphoma-derived exosomes have been reported to immunosuppress cytotoxic activity of NK cells by binding NKG2D receptors [50], it is possible the EVs obtained from the two cell lines examined in this work were lacking key modulatory proteins. Actual exosome protein expression is highly dependent on the cell of origin. While members of the tetraspanin family—CD9, CD63, and CD81—are often presented as “exosome markers”, this is not a binding condition. Early studies found CD63 to be low in exosomes compared to cells, while CD81 was enriched ten-fold [56]. Chronic Lymphocytic Leukemia (CLL) exosomes, for example, have been shown to be low in CD63 and CD81 [57] but high in CD20, HLA-DR, Hsp72, and XIAP. A study examining several B cell lymphoma cell lines found no CD9 expression and variable CD63 and CD81 levels [58]. Another common marker involved in exosome biogenesis is tumor susceptibility gene 101 (TSG101), whose levels have been known to vary widely in exosomes from plasma of different donors, as well as to be in the lumen of exosomes [59,60]. These findings highlight the importance of choosing markers for EV phenotyping carefully and recognizing the diversity in exosomes from different cellular origins. Characterization of the exosomes used in this study found their size to be within the accepted exosome range of 50–150 nm and in agreement with results from CLL exosomes noted to be 70–200 nm [57]. In this study, surface phenotyping revealed low CD63, CD9, and TSG101 expression, and moderate levels of CD81 and FasL. These lymphoma exosomes were also high in MHC class I markers but had no detectable TGF-β and MICA/B. Low exosomal levels of TGF-β and MICA/B may partly explain the observed inability of these vesicles to decrease NKG2D expression on NK cells [18,61].

Another factor may be found in the uptake capabilities of NK cells for these exosomes. Previous studies have shown that NK cells have limited capacity for EV internalization [47,62]. However, T cells also exhibit low uptake ability [63] and yet are still affected by EVs in a variety of capacities, including cytotoxicity, cytokine release, proliferation, and activation [40]. It should also be considered that K562 target cells are Fas negative; so, if NK cells are being modulated in their FasL expression, this would not elicit a change in the cytotoxicity assay [64].

While survivin did not have a significant effect on transcripts associated with NK cell activity, the functional proteins were decreased, perhaps through a post-translational mechanism. This may have an inhibitory influence on the ability of cancer-associated NK cells to eliminate tumor cells. The survivin-induced decrease in perforin and granzyme B may result in granules that are less effective, even if the degranulation process itself is unchanged.

Decreased cytokine levels after conditioning with extracellular survivin may hinder NK cell ability to communicate with other cells in the TME. TNF-α is an important inflammatory cytokine for the activation of neutrophils, macrophages, and lymphocytes, with roles in cell proliferation, differentiation, necrosis, apoptosis, and cytokine induction [65]. IFN-γ has a significant anti-tumor influence within the TME. It can slow the cell cycle of cancer cells, induce apoptosis, inhibit angiogenesis, and is a critical macrophage activating agent [66,67]. IFN-γ upregulates MHC class I molecules to promote antigen presentation and immune surveillance, promotes Th1 responses, and initiates a chemokine-directed recruitment of other immune cells [68]. An essential role of NK cells in the early anti-tumor response is the rapid production of IFN-γ. Therefore, survivin-induced reduction of IFN-γ may contribute to the suppression of cytotoxic T lymphocyte (CTL-) and NK-cell–mediated immune responses central to tumor immune escape. The immunomodulating effects of survivin that reduced IFN-γ are in line with the previous T cell study that reported skewed immune responses away from Th1 cell-mediated cytotoxicity [69]. Beyond its function in cell cycle and apoptosis, survivin is establishing roles in the extracellular environment of enhancing tumor progression and immune suppression. This interaction with the immune system is complex and warrants further exploration.

The finding that survivin can lower important proteins for NK cell function while not being able to elicit a noticeable decrease in the actual killing ability of the NK cells against target cells implies that survivin in the TME may be one component in a multifactorial inhibition of NK cells, with other entities also involved in the crippling of NK cell anti-tumor responses. Thus, anti-survivin therapies and anti-TEX therapies may be useful as part of a combination attack against cancer. Continuing to dissect and understand the contribution of exosomes to immune modulation is crucial for prevention of tumor escape, harnessing the patients’ own anti-tumor immune responses, and discovering novel therapies.

## Figures and Tables

**Table 1:** PCR primer sequences for cytokine and cytotoxic granule proteins.

Target Gene	NCBI Reference Sequence	Primer Sequence	Tm (°C)	Amplicon Size (bp)
IFN-γ	NM_000619.2	Sense 5′-CTGTTACTGCCAGGACCCAT-3′ Anti-sense 5′-GCATCTGACTCCTTTTTCGC-3′	59	412
TNF-α	NM_000594.3	Sense 5′-GTCCTCTTCAAGGGCCAAGG-3′ Anti-sense 5′-CAGACTCGGCAAAGTCGAGA-3′	57	258
Perforin	NM_005041	Sense 5′-TGGTGGACTACACCCTGGAA-3′ Anti-sense 5′-CACCTGGCATGATAGCGGAA-3′	57	561
Granzyme B	NM_004131.4	Sense 5′-GGCAGATGCAGGGGAGATCA-3′ Anti-sense 5′-TACAGCGGGGGCTTAGTTTG-3’	59	729
GAPDH	NM_002046.5	Sense 5′-ACGGATTTGGTCGTATTGGGCG-3′ Anti-sense 5′-CTCCTGGAAGATGGTGATGG-3′	60	212

Base pairs (bp), melting temperature (Tm), glyceraldehyde-3-phosphate dehydrogenase (GAPDH).

## Data Availability

All data is stored and is available upon request from NRW.
